# General and Electrophysiological Toxic Effects of Manganese in Rats following Subacute Administration in Dissolved and Nanoparticle Form

**DOI:** 10.1100/2012/520632

**Published:** 2012-05-01

**Authors:** Edina Horváth, Zsuzsanna Máté, Szabolcs Takács, Péter Pusztai, András Sápi, Zoltán Kónya, László Nagymajtényi, András Papp

**Affiliations:** ^1^Department of Public Health, University of Szeged Faculty of Medicine, Dóm tér 10., 6720 Szeged, Hungary; ^2^Department of Applied Chemistry, University of Szeged Faculty of Science and Informatics, Rerrich Béla tér 1., 6720 Szeged, Hungary

## Abstract

In an attempt to model occupational and environmental Mn exposures and their possible interaction, young male Wistar rats were exposed to Mn by oral administration in dissolved form (MnCl_2_ *·*4H_2_O, 14.84 and 59.36 mg/kg b.w.) and by intratracheal application of MnO_2_ nanoparticles (2.63 mg/kg b.w.). After 3 and 6 weeks oral, or 3 weeks oral plus 3 weeks intratracheal, exposure, general toxicological, and electrophysiological tests were done. Body weight gain was significantly reduced after 6 and 3 plus 3 weeks exposure, but the effect of the latter on the pace of weight gain was stronger. Organ weights signalized systemic stress and effect on lungs. Changes in evoked electrophysiological responses (cortical sensory evoked potential and nerve action potential) indicated that the 3 plus 3 weeks combined exposure caused equal or higher changes in the latency of these responses than 6 weeks of exposure, although the calculated summed Mn dose in the former case was lower. The results showed the importance of the physicochemical form of Mn in determining the toxic outcome, and suggested that neurofunctional markers of Mn action may indicate the human health effect better than conventional blood Mn measurement.

## 1. Introduction

It has been recognized in the last ca. 20 years that people's exposure to nanoparticles (NPs)s—let them be pollutants or components in nanotechnological products—has major influence on health. NPs are, by definition, particles which measure less than 100 nm in all three directions. In this size range, the surface-to-mass ratio becomes extremely high, meaning that this particle fraction (also called ultrafine dust) represents only a small mass fraction in any (environmental or occupational) dust sample but a high number of particles with a very high and reactive overall surface [1]. The small size also means that, once absorbed, NPs have extreme mobility within the (animal or human) organism and can reach all parts of it by crossing conventional barriers such as the alveolar or capillary wall. This, and the inflammogenic nature of NPs, is strongly influenced by their surface characteristics [2].

Concerning manganese (Mn), both fine and ultrafine particles containing this metal are likely generated in various phases of production and processing of Mn, from ore mining through steel casting and welding to the production of dry batteries; so that particle inhalation is a major way of—primarily occupational—exposure to this metal. The human nervous system effects of chronic Mn exposure are manifested by a state called manganism, a set of symptoms that is similar to Parkinson' disease and appears frequently in welders inhaling metal fumes [3]. Such exposure is typically job-related (but see [4]) while the general public might experience particulate Mn inhalation due to the use of methylcyclopentadienyl manganese tricarbonyl (MMT) as an antiknock petrol additive in some countries [5].

Manganese-induced Parkinsonism was, however, also observed in patients undergoing maintenance hemodialysis [6] or in inadvertent overdosing due to long-term ingestion of a health supplement containing high levels of Mn, which indicates that other physicochemical forms of Mn and other routes of exposure are also relevant to the health of the CNS. For geological reasons (e.g., in Greece [7]) or due to man-made pollution (such as improper disposal of used dry cells in Japan [8]) abnormally high Mn levels in the drinking water were observed, together with CNS symptoms of the affected population. In regions of the USA with high-Mn drinking water, loss of visual and verbal memory, typical for Mn-induced brain damage, was described [9]. The neurotoxic spectrum of Mn is variable: epileptic activity was observed in children following inhalational Mn exposure [4] or prolonged total parenteral nutrition [10], and alterations in EEG and evoked potential were seen in shipyard workers [11].

Based on the above literature data, indicating the relevance of both inhalational and oral exposure by Mn to nervous system effects, the present study was aimed at investigating the adverse neurofunctional effects in rats, caused by Mn in different physicochemical forms (by inhaled Mn NPs and by dissolved Mn taken up orally). Our goal was to create an experimental model reproducing complex human Mn exposure—including occupational and nutritional sources—more adequately, and to detect the effects by electrophysiological methods, the suitability of which was proven in previous works [12, 13].

## 2. Materials and Methods

### 2.1. Animals and Treatment

Young adult male Wistar rats (7 weeks old, body weight 200 ± 20 g) were obtained from the breeding centre of the university and were housed in a GLP-rated animal house (22 ± 1°C, 30–60% relative humidity, 12 h light/dark cycle with light on at 06:00), with free access to tap water and standard pellet (at start, there were 12 groups of 12 rats each; this number allowed for eventual losses during treatment, finally 8 rats per group were chosen randomly for evaluation).

Treatments, representing oral and inhalational exposure, were performed once daily, 5 times a week, and lasted altogether 3 or 6 weeks (see Table 1 for group codes, doses, and treatment times). The oral doses were based on an earlier work of us [14] where 14.84 and 59.36 mg/kg b.w. of MnCl_2_ were given by gavage for several weeks. The intratracheal dose of 2.63 mg/kg b.w. of MnO_2_ NPs was likewise tested in a previous experiment [12].

For oral application, manganese chloride (MnCl_2_ 4H_2_O; Reanal, Hungary; purity 99.5%) was dissolved in distilled water to 1 mL/kg b.w. administration volume and was given to the rats by gavage. The NPs used for intratracheal application consisted of MnO_2_, had 23.2 ± 3.3 nm diameter, and were synthesized at the Department of Applied Chemistry, University of Szeged by a technique combining sonication and hydrothermal treatment (see [13] for details). The NPs were suspended in 1% hydroxyethyl cellulose (HEC) dissolved in PBS (pH 7.4) to have a physiologically neutral vehicle in which unwanted surface interactions of the NPs were unlikely. The nanosuspension was instilled in the rats' trachea in brief diethyl ether anaesthesia (see [12] for details). The instilled volume was 1.0 mL/kg b.w. The summed dose (see Table 2) was calculated by adding the Mn° content of the daily administered volumes which were based on the daily body weights and the above-mentioned per kg doses.

### 2.2. General Toxicological Investigation

Body weight of the animals was measured before every treatment, for followup and for calculating the exact doses to be administered that day. Growth curves were plotted and the weight gain over the whole treatment period was determined. Following electrophysiological recording (the last phase of the investigations) the rats used were sacrificed by an overdose of urethane (the anesthetic used in electrophysiology), were dissected, and organ weights were measured. Relative organ weights, as a routine index of toxicity, were calculated on the basis of brain weight. This was chosen so (using the rationale described in [15]) because the brain weight was not significantly different among the groups while body weight, the other usual calculation basis, showed some significant changes (see Table 2).

### 2.3. Electrophysiological Investigation

Electrophysiological recording was done on the day following the last Mn administration. Preparation for recording, and the recording itself, was performed in urethane anaesthesia (1000 mg/kg i.p.) on 8 rats per group. The left hemisphere was surgically exposed (lidocaine spray was applied on the wounds) and spontaneous electrical activity (electrocorticogram, ECoG) was recorded from the primary somatosensory (SS), visual (VIS) and auditory (AUD) areas for 6 minutes using ball-tipped silver wire surface electrodes. From this, band spectrum according to the standard human EEG bands (delta to gamma [16]) was calculated. Then, evoked potentials (somatosensory, visual, and auditory) were recorded from the same sites by applying sensory stimuli in trains of 50.

Somatosensory stimulation was done by square pulses (3-4 V; 0.05 ms; 1, 2 and 10 Hz frequency) delivered through a pair of needles inserted into the contralateral whiskery skin. Visual stimulation was performed by flashes (1 Hz) of a high-luminance white LED placed to the contralateral eye of the rat. For acoustic stimulation, clicks (1 Hz) were applied to the contralateral ear through the hollow ear bar of the stereotaxic frame. Onset latency and duration of the EPs was measured after averaging the 50 individual records. From the tail nerve, compound action potential was recorded by inserting a pair of needle electrodes at the base of the tail for stimulation, and another pair 50 mm distally for recording. Conduction velocity was calculated from this distance and the onset latency of the action potential. Relative refractory period was measured by double stimuli with 1–10 ms interstimulus interval, from the extra delay of the second potential. The complete electrophysiological work was performed by means of the software Neurosys 1.11 (Experimetria Ltd., Budapest, Hungary).

During the whole course of the experiment, the principles of the Ethical Committee for the Protection of Animals in Research of the University were strictly followed.

### 2.4. Statistical Evaluation

The distribution of data was checked for normality by means of the Kolmogorov-Smirnov test. Data analysis was done by one-way ANOVA. Post hoc analysis of group differences was performed by LSD test, setting the probability level at *P* > 0.05.

## 3. Results

### 3.1. Body and Organ Weights

**Figure 1:**
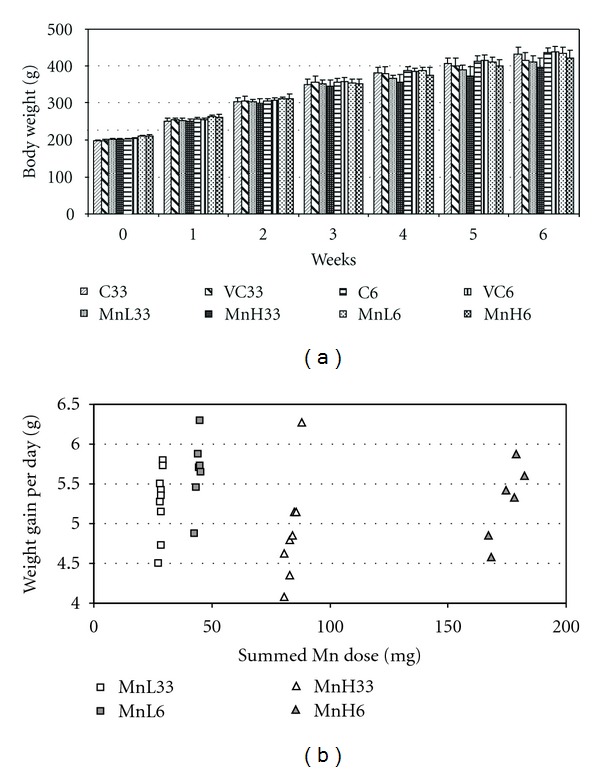
(a) Time course of the control and treated rats' weight gain in the 3 weeks oral + 3 weeks intratracheal and the 6 weeks oral treatment. Mean ± SD, *n* = 8. Significance marking omitted for clarity; for this, see Table 2. (b) Relationship of summed Mn dose (abscissa) and daily weight gain (ordinate) of individual rats belonging to the treatment groups given in the insert. Daily weight gain was calculated by dividing total weight gain with the length of treatment.

Oral Mn exposure caused only a light, nonsignificant reduction in the treated rats' body weight gain after 3 weeks, and after 6 weeks with the lower dose. Only after 6 weeks and the higher dose (MnH6) did weight gain show significant reduction. Where, however, intratracheal exposure followed the oral one (MnL33, MnH33) the reduction of weight gain was always significant. Compared to the corresponding controls, reduction of weight gain was somewhat stronger in the group MnH33 than in MnH6, although the calculated summed Mn dose was much lower in MnH33 than in MnH6 (Table 2). This suggested a potentially more efficient absorption of Mn from the airways than from the gastrointestinal tract. It is also noteworthy that in VC33 the weight gain reduction versus the corresponding untreated control was moderate (although present), indicating that the procedure of intratracheal administration itself (including repeated etherization) had no significant general toxic effect. The graph in Figure 1(a) shows the time course of weight gain (with an abrupt change at the beginning of intratracheal application of Mn NPs, but not of vehicle alone) while Figure 1(b) demonstrates that the individual rats' daily weight gain was more affected in the oral + intratracheal treatment groups in spite of the lower summed dose.

Among the relative organ weights, that of the lung, liver and adrenals showed significant changes (Table 3; the table also lists the absolute data of brain weight to show the basis of calculation). Lung weight was strongly affected by the intratracheal Mn treatment (MnL33, MnH33) and less strongly also by the intratracheal vehicle administration (VC33). The lungs excised from rats with intratracheal Mn exposure had typically an emphysematic appearance with visible dark spots of Mn deposition.

### 3.2. Electrophysiological Effects

There were no prominent changes in the spontaneous cortical activity. A trend of decreased power in the low-frequency (delta, and to a lesser extent, theta) bands, and increase in the fast beta2 and gamma bands was observed in all three cortical areas but without significance.

**Figure 2:**
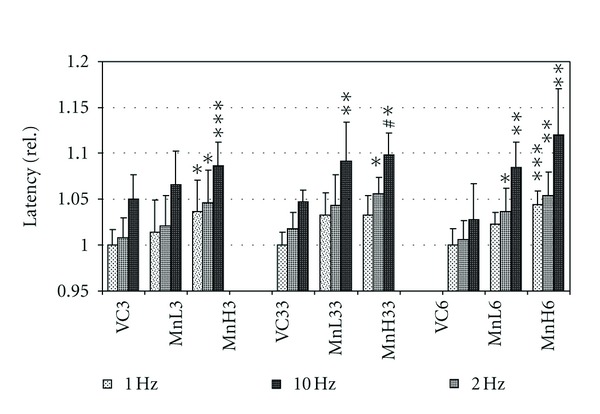
Latency of the somatosensory evoked potential in the control and treated rats, recorded with 1, 2, and 10 Hz stimulation frequency (see insert). Mean + SD, *n* = 8. The data displayed are relative, calculated to the value obtained from the vehicle-treated rats at 1 Hz frequency as basis of comparison.*, **, ***: *P* < 0.05, 0.01, 0.001 treated versus VC; ^#^, ^##^: *P* < 0.05, 0.01 MnL33 versus MnL6; °, °°, °°°: 2 Hz or 10 Hz stimulation versus 1 Hz, within one treatment group.

On the sensory EPs, Mn treatment generally caused a latency increase. As seen in Figure 2, SS EP latency was universally increased in the treated rats versus vehicle control. At the same time, the latency was also influenced by the frequency of stimulation, and this frequency-dependent lengthening was in the treated groups also typically more intense. Figure 2 also shows that latency increase in MnL33 was about equal to that in MnL6 at all stimulation frequencies, and also in MnH33 to MnH6 at 1 and 2 Hz stimulation; a relationship similar to that seen with the body weight gain (see Figure 1(b) and Table 2).

**Figure 3:**
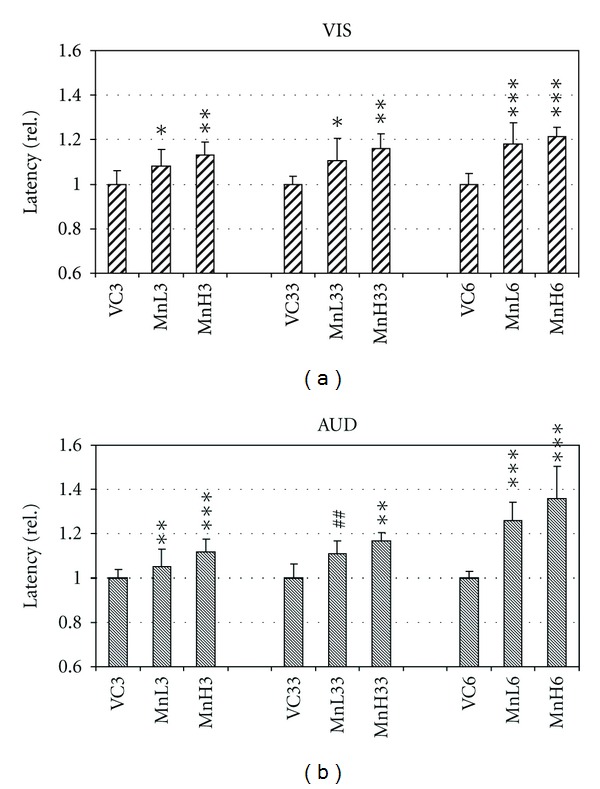
Latency of the visual (a) and auditory (b) evoked potentials in the control and treated rats. Mean + SD, *n* = 8. The relative data were calculated as given at Figure 2. *, **, ***: *P* < 0.05, 0.01, 0.001 treated versus VC; ^##^: *P* < 0.01 MnL33 versus MnL6.

The latency of VIS and AUD EPs was also lengthened by Mn exposure (Figure 3) but the relationship of the groups with 3 weeks oral + 3 weeks intratracheal versus 6 weeks oral treatment was not like in case of the SS EP. In case of all three sensory modalities, however, within one treatment variation the higher dose caused more lengthening of the latency: an indication of dose dependence, an exception being MnH33 versus MnL33 in case of SS EP. The duration of EPs showed no consistent alteration.

**Figure 4:**
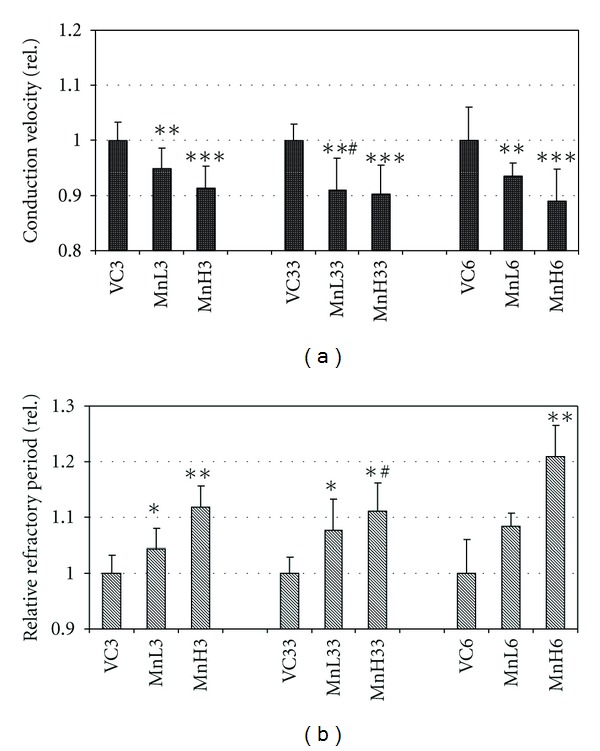
Changes of the nerve conduction velocity (a) and relative refractory period (b) in the tail nerve of the rats. Mean + SD, *n* = 8. The relative data were calculated as given at Figure 2. *, **, ***: *P* < 0.05, 0.01, 0.001 treated versus VC; ^#^: *P* < 0.05, 0.01 MnL33 versus MnL6.

The conduction velocity of the tail nerve was reduced in the treated rats. As seen in Figure 4(a), this effect was also dose dependent, and the effect in the group MnL33 was stronger than that seen in MnL6. The relative refractory period of the tail nerve (Figure 4(b)) was also significantly altered by Mn application.

## 4. Discussion

The most conspicuous result of the above experiments was the disproportionately strong effect seen with oral + intratracheal combined Mn exposure. That is, the change in body weight gain and in several electrophysiological parameters was more, or at least not less, expressed in the groups MnL33 and MnH33 (oral + intratracheal) than in MnL6 and MnH6 (oral only), although in the latter the applied summed dose was much higher (Table 2). This pointed to possible differences in the absorption and/or to qualitative differences in the toxicity of the two forms of Mn.

From the intestinal system, Mn is absorbed to only 10–15% [5, 17]. This is the physiological way of covering Mn demand so it is a regulated process where Mn overload leads to decreased absorption rate. From the airways, however, the absorption of NPs can be much more efficient. NPs translocate readily to extrapulmonary sites by a mechanism involving transcytosis (caveola formation) across epithelia of the respiratory tract into the interstitium, with subsequent access to the blood and distribution throughout the body [1]. Mn containing NPs can reach the brain from the blood through the capillary endothelial cells in the blood-brain barrier, and through the choroid plexuses [18]. Inhaled Mn absorbed this way is likely to reach its target sites before biliary excretion, the main mechanism of eliminating excess Mn from the organism [19]. In a more direct way, Mn-containing NPs enter the sensory nerve endings embedded in the epithelia of the airways (primarily, but not exclusively, in the olfactory mucosa) and migrate transsynaptically up to the brain [20].

Even if there was no tissue Mn level measurement in the present work, organ Mn load data of previous experiments with the same doses and routes of application [12–14] favor the reasoning that, on the basis of the observed toxic effects, inhaled NPs cause internal exposure more efficiently than ingested, dissolved Mn. An alternative, equally feasible explanation is that NPs had higher potency in inducing oxidative stress than dissolved Mn, keeping in mind that the surface chemistry of various oxide NPs is favorable for inducing oxidative stress [1].

Oxidative stress, first of all, contributed probably to the lung effects. Welding fumes containing Mn were reported to cause oxidative stress and inflammation [21]. In the liver, weight decrease was seen in rats with oral + intratracheal (MnL33, MnH33) exposure, but not in those receiving oral exposure only. A similar effect of NPs on the liver was seen also previously (with Mn NPs: Oszlánczi et al. [13]; but also with Cd NPs: Horváth et al. [22]). The dependence of the effect more on the nanoparticulate character than on the chemical composition is supported by literature data on in vivo liver damage related to oxidative stress in rats treated with intratracheal TiO_2_ NPs [23], and oxidative damage of in vitro human hepatic cells on exposure to ZnO NPs [24]. The decreased body weight gain in rats exposed to Mn NPs (oral + intratracheal treatment: groups MnL33 and MnH33) is also an indication of stress (as suggested in [25]) especially in combination with the increased weight of the adrenals in these groups (see Tables 2 and 3). Mn-induced systemic oxidative stress has been reported to act also in the brain and contribute to functional alterations [26]. Astrocytes were found to suffer oxidative damage on in vitro Mn exposure [27]. Others, however, found no connection between Mn neurotoxicity and ROS generation [28].

At the level of neurons, energetic shortage caused by Mn-dependent inhibition of mitochondrial complex II [29] and complex III [30] may lead to hypofunction of ion pumps and/or disturbed turnover of transmitters, and so to weakened propagation of the excitation. Increased frequency-dependent lengthening of the SS EP latency in treated versus control rats (see Figure 2) may be due to that. The mentioned mechanism of action was probably present not only on the cortical and subcortical level but along the whole sensory pathway because the pulse conduction velocity of the tail nerve (primarily of its fast myelinated axons, largely contributing to the recorded compound action potential and being especially sensitive to ATP depletion [31])—and, even more importantly, the relative refractory period, an indicator of the interaction of successive excitation processes—was also affected. Removal of glutamate from the synaptic cleft is slowed by the direct effect of elevated extracellular Mn level [32] and indirectly by the blocking effect of Mn-induced ROS on the high-affinity glutamate transporters [33]. Accumulation of excess glutamate at all transmission sites of the ascending sensory pathways may have resulted, after several weeks of Mn exposure, in receptor desensitization, another factor leading to increased EP latency [34]. The excitotoxic effect of excess glutamate cannot be ruled out either.

The results confirm the general and neurological toxicity of both forms of Mn applied, and emphasize the higher toxic potential of Mn-containing NPs as opposed to the dissolved form. Both of these forms, however, can cause human exposure, separately and simultaneously, and our present results potentially suggest that preceding (or concurrent) oral Mn burden may increase the sensitivity to inhaled Mn. This problem deserves further investigation, for example, in model systems similar to ours—partly because exposure from several sources or by several ways is a real-life possibility, and partly because Mn level in human biological samples is apparently not a reliable indicator of CNS damage [35] and, not less importantly, is not sensitive to the physicochemical form of the absorbed Mn. So, eventual development of neurofunctional biomarkers, based for example on the electrophysiological effects examined in this work, may be of importance—primarily in contributing to sensitivity while Mn specificity will have to be provided by other, probably chemical, markers.

## Figures and Tables

**Table 1:** Treatment scheme with group codes, doses and treatment times.

Group code	Treatment and dose
C3	3 weeks, untreated
VC3	3 weeks, distilled water, orally
MnL3	3 weeks 14.84 mg/kg b.w. MnCl_2_, orally
MnH3	3 weeks 59.36 mg/kg b.w. MnCl_2_, orally
C33	6 weeks, untreated*
VC33	3 weeks, distilled water, orally + 3 weeks HEC intratracheally
MnL33	3 weeks 14.48 mg/b.w. kg MnCL_2_, orally + 3 weeks MnO_2_ NPs 2.63 mg/kg b.w. intratracheally
MnH33	3 weeks 59.36 mg/b.w. kg MnCL_2_, orally + 3 weeks MnO_2_ NPs 2.63 mg/kg b.w. intratracheally
C6	6 weeks, untreated*
VC6	6 weeks, distilled water, orally
MnL6	6 weeks 14.84 mg/kg b.w. MnCl_2_, orally
MnH6	6 weeks 59.36 mg/kg b.w. MnCl_2_, orally

*C33 and C6 were two different groups of rats.

**Table 2:** Body weight gain of the rats during the treatment period.

Group	Body weight gain (g)	Summed Mn dose (mg Mn°/animal)
C3	161.7 ± 14.4	—
VC3	149.9 ± 11.2	—
MnL3	149.2 ± 13.9 (99.6%)	17.94 ± 0.52
MnH3	143.7 ± 23.6 (95.9%)	71.58 ± 2.93
C33	234.2 ± 19.7	—
VC33	218.4 ± 20.9	—
MnL33	210.9 ± 17.2* (96.5%)	28.46 ± 0.55
MnH33	195.2 ± 27.6^∗∗#^ (89.4%)	83.42 ± 2.95
C6	234.2 ± 25.0	—
VC6	235.2 ± 15.3	—
MnL6	226.1 ± 17.3 (96.2%)	44.55 ± 1.00
MnH6	211.0 ± 19.3^#^ (89.7%)	178.43 ± 8.53

For group codes, see Table 1. The data represent group averages (mean ± SD, *n* = 8) of the difference between body weight on day 0 (before the first Mn administration) and the day of sacrifice.

*, **: *P* < 0.05, 0.01 versus C; ^#^: *P* < 0.05 versus VC.

Percent values in parentheses represent the weight gain in the Mn-treated groups compared to that in the corresponding VC group.

**Table 3:** Relative organ weights of the lungs, liver, and adrenals, and the absolute brain weights used as calculation basis.

Groups	Brain absolute weight (g)	Relative organ weights		
		Lungs	Liver	Adrenals
C3	1.989 ± 0.061	0,170 ± 0.009	1,856 ± 0.104	0,0070 ± 0.0010
VC3	1,999 ± 0.072	0,200 ± 0.021*	1,606 ± 0.103*	0,0085 ± 0.0027
MnL3	2,038 ± 0.095	0,167 ± 0.017^#^	1,837 ± 0.207^#^	0,0070 ± 0.0008
MnH3	1,974 ± 0.111	0,224 ± 0.055^∗X^	1,840 ± 0.299	0,0081 ± 0.0011^X^
C33	2,076 ± 0.061	0,169 ± 0.020	1,534 ± 0.101	0,0061 ± 0.0013
VC33	2,092 ± 0.067	0,214 ± 0.009**	1,563 ± 0.113	0,0062 ± 0.0007
MnL33	2,053 ± 0.043	0,391 ± 0.056^∗∗∗###^	1,626 ± 0.133	0,0078 ± 0.0014^∗#^
MnH33	2,106 ± 0.077	0,372 ± 0.018^∗∗∗###^	1,450 ± 0.075^#X^	0,0073 ± 0.0014*
C6	2,127 ± 0.047	0,151 ± 0.013	1,512 ± 0.102	0,0057 ± 0.0006
VC6	2,092 ± 0.028	0,154 ± 0.013	1,443 ± 0.088	0,0064 ± 0.0014
MnL6	2,124 ± 0.076	0,149 ± 0.011	1,546 ± 0.081	0,0070 ± 0.0009*
MnH6	2,103 ± 0.060	0,165 ± 0.022	1,430 ± 0.106	0,0063 ± 0.0008

For group codes, see Table 1; for the calculation, see Section 2. The data are mean ± SD (*n* = 8).

*, **, ***: *P* < 0.05, 0.01, 0.001 versus C; ^#^, ^###^ *P* < 0.05, 0.001 versus VC; ^X^: *P* < 0.05 MnH versus MnL.
